# Sex differences in alcohol dehydrogenase levels (ADH) and blood ethanol concentration (BEC) in Japanese quail

**DOI:** 10.1016/j.psj.2022.101790

**Published:** 2022-02-18

**Authors:** Shannon E. Eaton, Julia E. Jagielo-Miller, Mark A. Prendergast, Chana K. Akins

**Affiliations:** ⁎ Arizona State University, Department of Psychology, Tempe, AZ, USA; † University of Kentucky, Department of Psychology, College of Arts & Sciences, Lexington, KY, USA

**Keywords:** alcohol, quail, alcohol dehydrogenase, blood ethanol concentration, rate of absorption, rate of disappearance

## Abstract

Ethanol is one of the most widely used and abused drugs. Following ethanol consumption, ethanol enters the bloodstream from the small intestine where it gets distributed to peripheral tissues. In the bloodstream, ethanol is cleared from the system by the liver. The primary metabolism of ethanol uses alcohol dehydrogenase (**ADH**). In mammals, females appear to have higher ADH activity in liver samples than males. The purpose of the first experiment was to analyze sex differences in ADH levels following 12 d of ethanol administration (i.e., water or 2 g/kg) in male and female quail. Following the last daily treatment of ethanol, quail were euthanized, their livers were extracted, and ADH was analyzed in liver homogenate samples. Results showed that female quail had higher ADH levels, heavier livers, and a greater liver to body weight ratio than male quail. In a second experiment, we aimed to develop a blood ethanol concentration (**BEC**) profile for both male and female quail. Quail were administered 0.75 or 2 g/kg of ethanol and blood was collected at 0.5, 1, 2, 4, 6, 8, 12, 24 h after gavage administration. Blood ethanol concentration was analyzed using an Analox. We found that quail had a fairly rapid increase in BECs followed by a steady and slow disappearance of ethanol from the blood samples. Female quail had a lower peak of ethanol concentration and a smaller area under the curve (**AUC**) than male quail. The current research suggests that higher ADH levels in female quail may be responsible for increased metabolism of ethanol. In general, quail appear to eliminate ethanol more slowly than rodents. Thus, as a model, they may allow for a prolonged window with which to investigate the effects of ethanol.

## INTRODUCTION

Ethanol is a central nervous system depressant and it is consumed for its psychoactive effects (Hendler et al., 2011). When consumed, it is readily absorbed into the bloodstream from the small intestine and is distributed throughout the body into peripheral tissues (Levitt et al., 1997). There are a few factors that influence the rate at which ethanol is absorbed, including the amount of ethanol consumed, body composition, gastric emptying, and enzymatic activity (for review see, Crabb et al., 1987; Frezza et al., 1990). The amount of ethanol consumed affects absorption such that higher doses diffuse across membranes more readily. Additionally, the rate at which the stomach is emptied affects the rate at which ethanol is absorbed such that faster emptying results in higher blood ethanol concentration (**BEC**) (Holt, 1981). The distribution of ethanol may also be influenced by body composition, specifically by total body water. The same dose of ethanol may vary drastically in distribution due to fat and water variations in the body, even in individuals who weigh the same amount (Davies and Bowen, 1999).

Following absorption, ethanol is mainly metabolized in the liver by 2 pathways (Cederbaum, 2012). The primary pathway uses alcohol dehydrogenase (**ADH**) which breaks down ethanol by catalyzing the oxidation of ethanol into acetaldehyde, a toxic metabolite. Acetaldehyde is associated with many unpleasant effects including facial flushing and nausea. Acetaldehyde is further broken down by aldehyde dehydrogenase (**ALDH**) into acetate, which can be further broken down into acetyl CoA. These pathways account for approximately 90% of the metabolism of ethanol (Cederbaum, 2012). The main site for ethanol metabolism is the liver where ADH levels are highest compared to other tissues (Boleda et al., 1989).

In both humans and rodents, there appears to be a sex difference in ADH levels that may affect the metabolism of ethanol as reflected in BECs. In humans, men have higher gastric ADH activity than women resulting in men having lower peak BECs when alcohol is consumed orally (Frezza et al., 1990; Seitz et al., 1993). However, women have higher ADH activity in the liver compared to men (Mezey, 2000) which may result in faster elimination of ethanol resulting in lower BECs (Dettling et al., 2007). Similar to humans, male mice have more gastric ADH activity than female mice (Desroches et al., 1995) and female mice and rats have higher hepatic ADH than males (Kishimoto et al., 2002; Simon et al., 2002).

These sex differences in ADH activity may be related to differences in ethanol elimination. In mice, Kishimoto et al. (2002) found a sex difference in the elimination of ethanol but other research found no difference (Livy et al., 2003; Lopez et al., 2003). Rats show a similar pattern as mice such that female rats have more ADH activity (Simon et al., 2002; Quintanilla et al., 2007) and faster ethanol elimination compared to males (Robinson et al., 2002). Taken together, it follows that sex differences observed in ADH activity may be associated with a faster ethanol elimination rate from the blood.

Levels and activity of ADH also appear to be affected by ethanol administration. Drosophila larvae fed an ethanol diet had a two-fold increase in ADH levels compared to larvae fed a control diet (McKechnie and Geer, 1984). In zebrafish, ADH activity following acute ethanol exposure follows an inverse U pattern based on the dose (Tran et al., 2015). Similarly, in mice, both ADH activity and content were affected by dose and followed a similar inverse U pattern (Haseba et al., 2012). Chronic administration of ethanol in rats resulted in a gradual increase of ADH, peaking at 26 wk before decreasing (Dajani et al., 1963). Taken together, ADH levels and activity appear to be higher following treatment with ethanol.

Research on ADH in quail has been limited to the development of ADH classes and their expression in quail in in vitro studies. Quail have 4 classes of ADH enzymes that share some similarities with mammalian ADH enzymes (Nussrallah et al., 1989). ADH develops similarly between males and females early in ontogeny, but adult levels mainly differ in the class that is dominantly expressed, with males expressing the ADH1, ADH2, and ADH3 classes and females predominantly expressing the ADH3 class. The elimination rate of ADH in quail is similar to the human class 1 ADH enzyme (Kaiser et al., 1990).

Research has previously established BEC temporal profiles in rats and mice (Livy et al., 2003). However, there is relatively little known about the BEC profile in birds. One study examined BECs in fruit-eating birds (Eriksson and Nummi, 1982) by injecting fruit-eating birds (waxwings, starlings, and bullfinches) with 1 or 2 g/kg ethanol intraperitoneally (**ip**) and observing the rate of ethanol elimination. They found that the 2 g/kg dose of ethanol was eliminated in about 2 h in waxwings, about 3 h in starlings, and about 13 h in greenfinches (Eriksson and Nummi, 1982). Another study examined a more controlled approach by injecting ethanol (i.e., 2 and 3 g/kg ip) in finches (Olson et al., 2014). They found that finch BECs rose rapidly within the first 30 min and remained elevated for at least 3 h. However, no studies have examined BECs across time or developed a BEC profile in birds.

The objectives of the current research were to examine ADH levels in male and female quail since these enzymes may play a role in the development of AUD (Experiment 1) and develop a BEC profile in quail (Experiment 2). Quail were chosen because previous research has shown that they may be a good model to study motivational properties of drugs of abuse (Mills et al., 1997; Akins and Geary, 2008; Rosine et al., 2009; Bolin et al., 2012). However, there is currently no research examining ethanol pharmacokinetics in quail. First, we hypothesize that there will be a sex difference in ADH because, in unpublished studies, female quail appeared to metabolize ethanol more quickly than male quail. Therefore, we predict that female quail will have higher levels of ADH than males. Second, we hypothesize that ethanol treatment will have an effect on ADH levels. Finally, similar to previous research that developed a BEC temporal profile for rats and mice (Livy et al., 2003), the current research aimed to create a BEC profile in quail. This BEC profile will allow for a better understanding of the pharmacokinetics of blood ethanol levels in quail following an oral administration. We predict a dose-dependent difference in peak BECs with the higher dose of ethanol resulting in increasing levels of BEC and maintenance of high levels of BECs across time relative to a low dose of ethanol. Additionally, we predict that female quail will metabolize ethanol more quickly than male quail and that this may contribute to different BEC profiles.

## MATERIALS AND METHODS

### Experiment 1

#### Subjects

Adult male (N = 18) and female (N = 23) Japanese quail (*Coturnix japonica*) were used in this experiment. Fertilized eggs were supplied by GQF Manufacturing (Savannah, GA) and once hatched, birds were raised at the University of Kentucky. All quail were housed under a 16:8 L:D cycle and had ad lib access to food and water. Quail were kept in mixed-sex brooders until 28 d post-hatch, and then males were individually housed, and females remained group housed. Before starting the experiment, all quail were individually housed and habituated to the colony for at least 2 wk. Quail were 5 to 6 mo old at the start of the experiment and at that time they were randomly assigned to receive water (N = 22; 12 males and 10 females) or ethanol (N = 19; 6 males and 13 females) repeatedly for 12 d. All procedures were conducted in accordance with the National Institutes of Health Guide for the Care and Use of Laboratory Animals, and experimental procedures were approved by the Institutional Animal Care and Use Committee at the University of Kentucky.

#### Drugs

Water or ethanol (25% w/v) was administered by gavage once daily for 12 d.

#### ELISA Procedure

Twenty-four hours after the last administration, quail were euthanized by rapid decapitation and trunk blood was collected in heparinized tubes. The tubes were then centrifuged at 1,500 RPM (21,890 × *g*) for 15 min, and the plasma separated. Livers were extracted and washed in PBS before being frozen in isopentane which was cooled by dry ice. All samples were stored at −80°C until assayed. In preparation for the ELISA, livers were thawed, rinsed, and weighed. Then 200 mg of the liver was added to 500 uL of phosphate-buffered saline (**PBS**) and homogenized in a glass tube homogenizer on ice. The liver homogenate was then sonicated. The samples were centrifuged at 1,500 RPM (21,890 × *g*) for 10 min, the lipid layer was then removed, and the samples respun at 1,500 RPM (21,890 × *g*) for 15 min before the supernatant was collected.

Alcohol dehydrogenase (**ADH**) levels were measured in duplicate via an enzyme-linked immunoassay (**ELISA**) kit (MyBioSource; MBS743834, San Deigo, CA) according to the manufacturer's instructions. Briefly, 100 uL of samples and standards were transferred to assigned wells, followed by 50 uL of enzyme conjugate. The plate (96-well micro-titer) was then allowed to incubate for 1 h at 37°C. After incubation, the wells were washed with the provided solution 5 times, and 50 uL of each substrate was added and allowed to incubate for 20 min at 37°C. A stop solution was then added and optic densities were immediately analyzed at 450 nm using a Beckman Coulter DTX 880 Multimodal Detector (Lagerhausstrasse, Austria) and Beckman Coulter Multimode Detection Software (v.20.0.12). Results were determined using a 4-parameter logistic standard curve analysis within SigmaPlot version 14 (Systat Software, Inc., San Jose, CA).

### Experiment 2

#### Subjects

The subjects in this experiment were 5 to 6-mo-old male (N = 23) and female (N = 25) Japanese quail (*Coturnix japonica*). Fertilized eggs were purchased from GQF Manufacturing and hatched and raised at the University of Kentucky. Seven birds (4 females and 3 males) were provided as adults from Centre College. All animal care was the same as in Experiment 1.

#### Blood Ethanol Concentration Procedure

Based on previous research (Gauvin et al., 2000), quail were randomly assigned to receive a 0.75 (9 males and 14 females) or 2.0 g/kg (14 males and 11 females) dose of ethanol (Pharmaco-Aaper, Brookfield, CT) by gavage (25% w/v alcohol in tap water). Blood was collected from the brachial (i.e., wing) vein at 30 min, 1, 2, 4, 6, 8, 12, and 24 h after gavage administration. These time points were chosen to help create a complete and comprehensive metabolism curve. Previous research suggested that the metabolism and elimination of ethanol may have been substantially longer in birds than in rodents (Olson et al., 2014). Blood samples were centrifuged at 1,500 RPM (21,890 × *g*) for 5 min, and plasma separated and stored at −80°C until assayed.

Plasma was used to measure BECs using an Analox AM1 instrument (Analox Instruments, London, UK). The Analox instrument catalyzes the oxidation of ethanol such that the ethanol concentration is based on the maximum rate of oxygen consumption. BEC analysis was used to determine various pharmacokinetic properties including peak levels and area under the curve.

### Statistical Analysis

Sex differences and the effect of ethanol on ADH levels and liver weights were analyzed with a 2 × 2 (sex × treatment) analysis of variance (**ANOVA**) using SPSS (version 27, IBM Corp., Armonk, NY).

To analyze the pharmacokinetics of alcohol, absorption and elimination were analyzed as the slope to or from peak using a simple slope analysis in GraphPad (version 8.0.0 for Windows, GraphPad Software, San Diego, CA). Peak BEC was determined as the highest measured value. Additionally, the area under the curve was analyzed by Prism using GraphPad to assess and compare between groups. Prism calculates the AUC by using the trapezoid rule. Difference over time was analyzed using a repeated-measures ANOVA with sex (male and female) and ethanol dose (0.75 and 2.0 g/kg) as between factors and time (30 min, 1, 2, 4, 6, 8, 12, and 24 h) as the within-subjects factor to determine any differences in BECs in SPSS (IBM Corp., version 27). When the assumption of sphericity was violated, the Greenhouse-Geisser correction was used, with α set at < 0.05. Post hoc analyses were conducted using Tukey tests.

## RESULTS

### Experiment 1

#### ADH Levels and Ethanol Treatment

**Figure 1:**
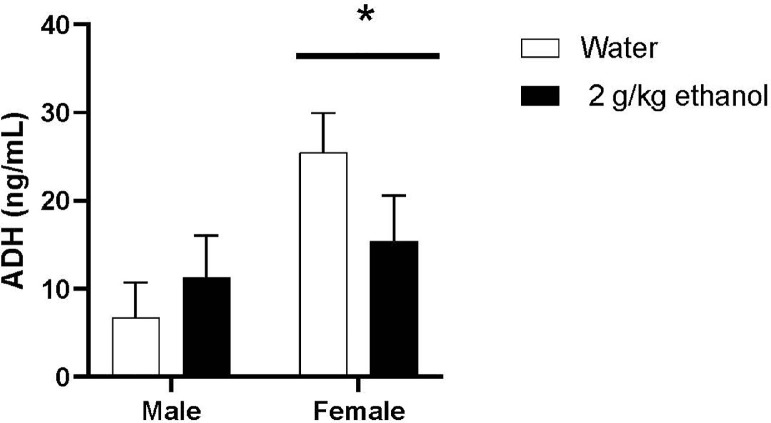
Mean (±SEM) alcohol dehydrogenase (ADH) levels (ng/mL) for male and female quail that received water or 2 g/kg ethanol once a day for 12 d. * indicates a significant difference from males, *P* < 0.05.

ADH levels (ng/mL) in male and female livers are shown in Figure 1. Female quail (*M* = 20.436, *SEM* = 3.413) had higher ADH levels than male quail (*M* = 8.99, *SEM* = 3.114) as revealed by a main effect of sex, *F*(1,27) = 6.137, *P* = 0.020, η2 = 0.166. There was no main effect of treatment *F*(1,27) = 0.354, *P* = 0.557, η2 = 0.013, and no significant interaction between sex and treatment *F*(1,27) = 2.476, *P*=0.127, η2 = 0.084.

#### Liver Weights

**Figure 2:**
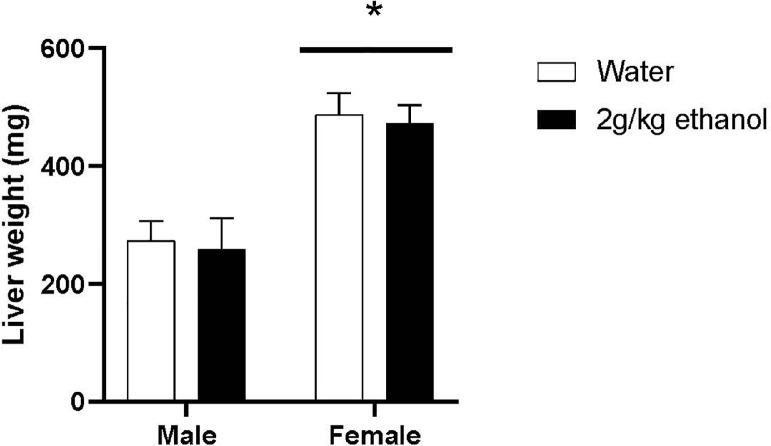
Mean (±SEM) liver weights for male and female quail that received water or 2 g/kg ethanol once a day for 12 d. * indicates a significant difference from males, *P* < 0.05.

Figure 2 shows the overall weight of the livers in grams. An ANOVA revealed a significant main effect of sex for liver weight, *F*(1,37) = 29.747, *P* < 0.001, η2 = 0.446, with female quail (*M* = 479.688, *SEM* = 24.037) having significantly heavier livers than male quail (*M* = 266.158, *SEM* = 30.902). However there was no main effect of treatment, *F*(1,37) = 0.120, *P* = 0.731, η2 = 0.003, nor an interaction between sex and treatment, *F*(1,37) = 0.0, *P* = 0.999, η2 = 0.0.

#### Ratio of Liver Weight to Total Body Weight

**Figure 3:**
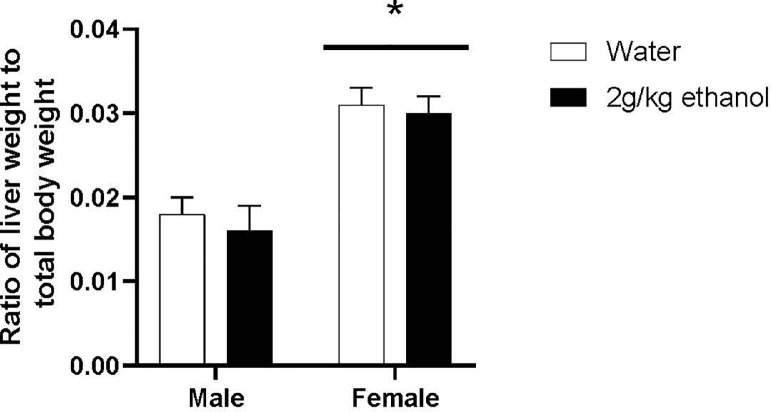
Mean (±SEM) liver to total body weight ratios for male and female quail that received water or 2 g/kg ethanol once a day for 12 d. * indicates a significant difference from males, *P* < 0.05.

Figure 3 shows liver weights as a ratio of total body weight. An ANOVA revealed a significant main effect of sex in the ratio of liver weight to body weight, *F*(1,37) = 34.439, *P* < 0.001, η2 = 0.482, with female quail (*M* = 0.031, *SEM* = 0.001) having a significantly greater liver weight to body weight ratio than male quail (*M* = 0.017, *SEM* = 0.002). However, there was no main effect of treatment, *F*(1,37) = 0.595, *P* = 0.445, η2 = 0.016, nor an interaction between sex and treatment, *F*(1,37) = 0.0, *P* = 0.984, η2 = 0.0.

### Experiment 2

#### BEC Profile

**Figure 4:**
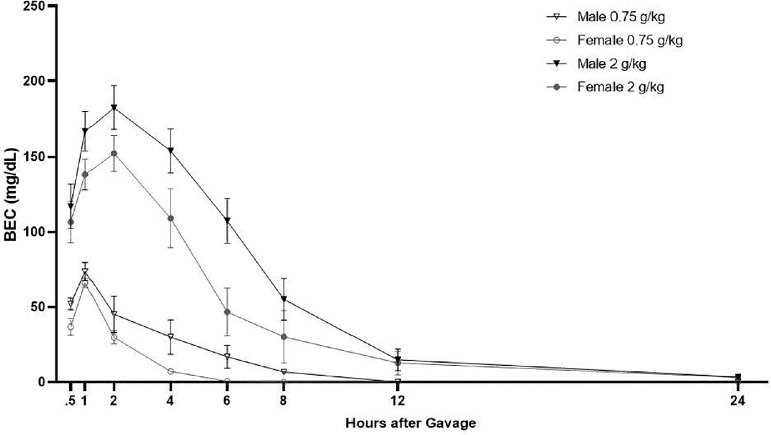
Mean blood ethanol concentration over 24 h for male and female quail following a gavage of 0.75 g/kg or 2 g/kg ethanol.

Figure 4 shows the average BEC at each time point following a gavage of either 0.75 or 2 g/kg ethanol. BECs rose quickly reaching peak levels around 1 (0.75 g/kg) or 2 h (2 g/kg) after gavage, followed by a slow reduction in BECs for the next few hours depending on the dose. A RM ANOVA revealed a main effect of time, [*F*(3,36) = 25.08, *P* < 0.001, η2 = 0.695], a main effect of treatment [*F*(1,11) = 16.702, *P* = 0.002, η2 = 0.603] and a time by treatment interaction [*F*(3,36) = 5.472, *P* = 0.003, η2 = 0.332]. Post hoc analyses revealed quail treated with 0.75 g/kg had BECs that were significantly different from the first time point (i.e., 30 min) at 8, 12, and 24 h, *P* < 0.05. They also had higher BECs at 60 min compared to 6, 8, 12, and 24 h following ethanol administration, *P* < 0.05. Quail treated with 2 g/kg had BECs that were significantly different from the first time point (i.e., 30 min) at 2, 4, 8, 12, and 24 h, *P* < .005. BECs at 60 min were significantly different from time points 6 to 24 h, and their BECs at 2 h were significantly greater than 30 min and 4 to 24 h, *P’s* < 0.05. No main effect of sex or an interaction with sex was evident.

#### Peak BEC Levels

**Figure 5:**
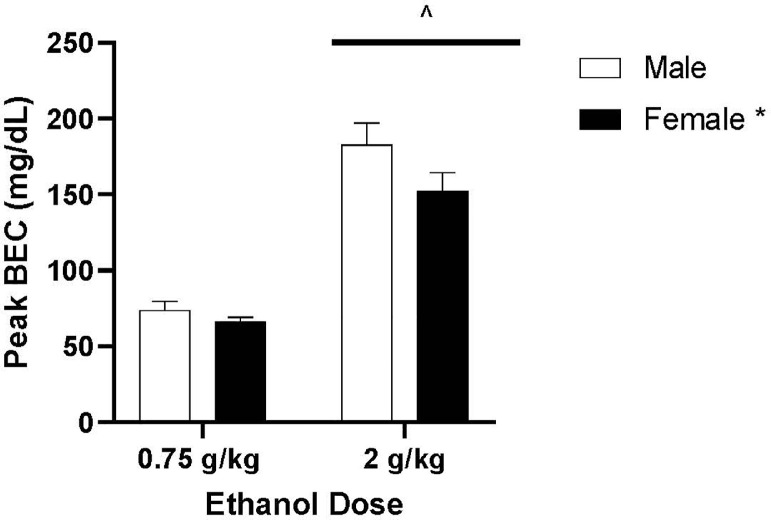
Mean peak blood ethanol content (±SEM) for male and female quail gavaged with 0.75 g/kg or 2 g/kg ethanol. ^ indicates a significant difference from 0.75 g/kg. * indicates a significant difference from males

Figure 5 shows the average peak BEC reached by male and female quail treated with 0.75 or 2 g/kg ethanol. A 2 × 2 ANOVA revealed a main effect of sex [*F*(1,44) = 4.032, *P* = 0.049, η2 = 0.060] and a main effect of dose [*F*(1,44) = 262.276, *P* < 0.001, η2 = 0.856] on peak BEC levels. Male quail reached a higher peak BEC compared to females. Quail treated with 0.75 g/kg ethanol (*M* = 69.878, *SEM* = 5.443) had lower peak BECs than quail treated with 2 g/kg ethanol (*M* = 191.051, *SEM* = 5.133). There was no interaction between sex and ethanol dose [*F*(1,44) = 0.026, *P* = 0.872, η2 = 0.001].

#### Area Under the Curve

**Figure 6:**
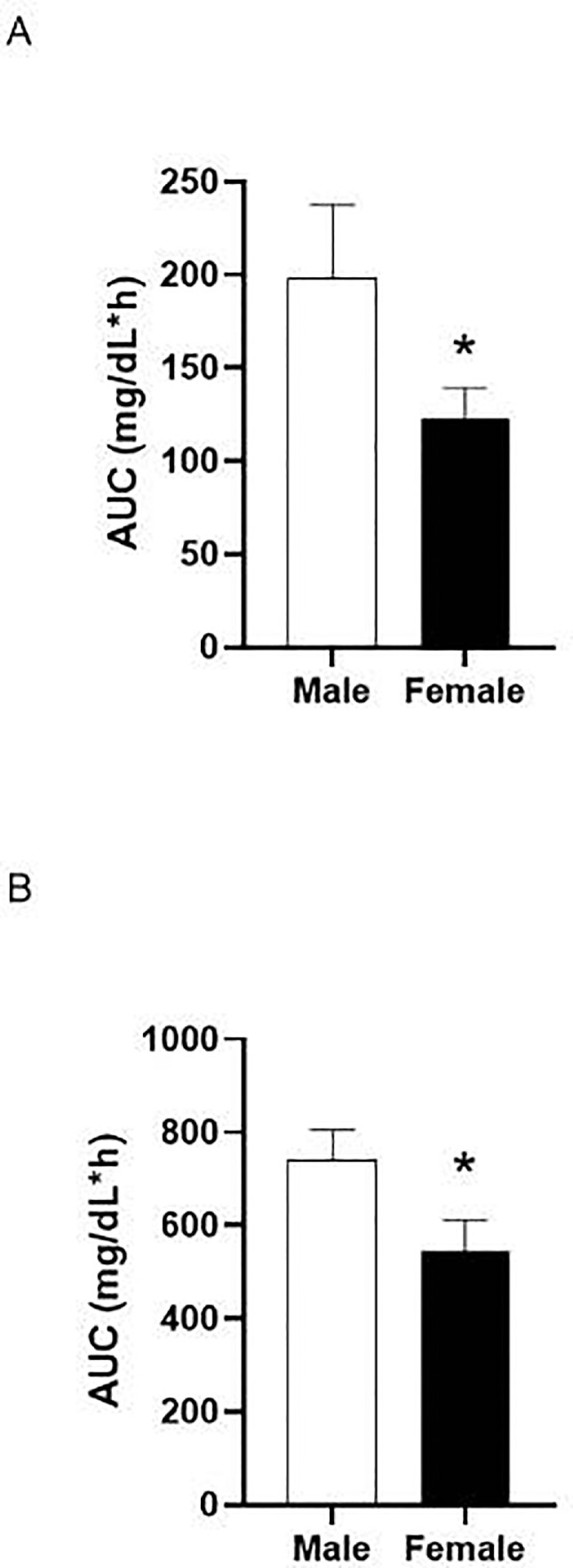
Mean area under the curve (±SEM) for male and female quail gavaged with 0.75 g/kg (A) or 2 g/kg (B) * indicates a significant difference from male quail.

Figure 6 shows the average area under the curve (**AUC**) for male and female quail treated with 0.75 (A) and 2 g/kg (B). Student's *t* test revealed a sex difference for quail treated with 0.75 g/kg [t(144) = 2.004, *P =* 0.047, R^2^ = 0.0271]. When treated with 0.75 g/kg, female quail (*M* = 122.1, *SEM* = 17.2) had a smaller AUC than male quail (*M* = 198.3, *SEM* = 39.25). Additionally there was a sex difference for quail treated with 2 g/kg [t(96) = 2.107, *P = 0.038,* R^2^ = 0.0442], indicating that female quail (*M* = 544.2, *SEM* = 68.0) had a smaller AUC than male quail (*M* = 741.2, *SEM* = 64.2).

#### Ethanol Absorption

Ethanol absorption was measured as the slope of the BECs from the first time point (i.e., 30 min) to 60 min. The average amount of change in BEC was 42.91 for males and 58.59 for females treated with 0.75 g/kg ethanol. For quail treated with 2 g/kg of ethanol, the average amount of change in BEC to peak was 99.82 for males and 63.34 for females. A comparison of the simple slopes failed to reveal a main effect of sex for the 0.75 g/kg ethanol-treated quail [*F*(1,36) = 0.6360, *P* = 0.4304] and the 2 g/kg ethanol-treated quail [*F*(1,38) = 0.4334, *P* = 0.5143].

#### Ethanol Elimination

Ethanol elimination was measured as the slope from peak to the first point following the peak BEC. The average amount of change in slope for BECs following the peak was −28.48 for males and −36.22 for females treated with 0.75 g/kg ethanol. For quail treated with 2 g/kg ethanol, the average amount of change in BEC following peak was −14.36 for males and −21.61 for females. A comparison of the simple slopes failed to reveal an effect of sex for 0.75 g/kg ethanol-treated quail, [*F*(1,36) = 0.3679, *P* = 0.5480] and the 2 g/kg ethanol-treated quail [*F*(1,24) = 1.541, *P* = 0.2264].

## DISCUSSION

The findings of the current experiments indicated that overall, sex differences were found in ADH levels and liver weights such that female quail had greater ADH levels, greater overall liver weights, and a greater liver to body weight ratio than male quail. However, there was no effect of ethanol treatment on any of the ADH-related measures. Additionally, we developed a BEC temporal profile for male and female quail and observed some sex differences within the BEC profile. Specifically, female quail had lower peak BECs and a smaller AUC compared to male quail.

The findings of Experiment 1 extend previous work that examined ADH in quail by quantifying ADH in the male and female liver (Nussrallah et al., 1989). Previous work was focused on the development of ADH classes and their expression in male and female quail The current research extended their findings by finding that ADH levels were greater in female quail compared to male quail. In addition, previous research has shown that females across species have more hepatic ADH compared to males (Kishimoto et al., 2002; Quintanilla et al., 2007). The current research found similar results in that female quail had higher ADH levels than males. Taken together, the sex difference in ADH levels appears to be conserved in quail.

Similar to previous research, we found a sex difference in both liver weights and liver to body weight ratios. Female quail had much heavier livers than male quail. These findings are in agreement with previous quail research which found that females have heavier livers than male quail (Tserveni‐Gousi and Yannakopoulos, 1986; Toelle et al., 1991; Selim et al., 2006). Liver weight to body weight ratios in birds vary across species but those with higher liver weight to body weight also have faster ethanol elimination (Eriksson and Nummi, 1982).

The current research failed to find an increase in ADH levels following repeated ethanol treatment (i.e., 12 d). This lack of finding may have been due to the length of time required to observe any change in ADH levels. ADH studies that have found an increase in ADH levels following ethanol treatment found an increase following chronic or continuous exposure (Mirone, 1965; Tran et al., 2015). For example, ADH activity was elevated in rats pretreated with ethanol for 21 d compared to non-pretreated controls (Mirone, 1965). In another study, eight weeks of exposure to ethanol vapor decreased ADH expression in the livers of rats (Mouton et al., 2016). Similarly, Zebrafish exposed to ethanol continuously for 22 d had an increase in ADH activity, but fish that only received ten days of repeated exposure did not show a change in ADH activity compared to controls (Tran et al., 2015). Therefore, the lack of increase in ADH levels in the ethanol-treated quail of the current study appears to be in line with previous findings which suggest that an extended period of ethanol administration may be required.

The current study failed to reveal any changes in liver weight or the liver to body weight in the ethanol-treated group. Rats pretreated with ethanol for 21 d had heavier liver weights compared to non-pretreated rats (Mirone, 1965). Furthermore, mice that were allowed 1 mo ad lib access to ethanol as the sole drinking fluid had heavier livers and a greater liver to body weight ratio (Okuda et al., 2018). Liver volume is strongly correlated to the elimination rate of ethanol in rats (Lumeng et al., 1979). Thus, the failure to observe ethanol-induced changes in liver properties may be due to the same reason we did not observe changes in ADH levels such that the length of ethanol treatment may have needed to be longer to observe these differences.

The findings of Experiment 2 extend previous ethanol pharmacokinetics research by developing a BEC profile for a bird species. In a previous study, finches that received ethanol (2 or 3 g/kg) ip had relatively high BECs and BECs remained high 3 h later (Olson et al., 2014). Eriksson and Nummi (1982) found that a 2 g/kg dose of ethanol administered ip was eliminated in about 2 h in waxwings, about 3 h in starlings, and about 13 h in greenfinches. In comparison, the current study found that the BECs of quail were dose-dependent and remained relatively high until about 6 to 8 h after gavage of ethanol.

Experiment 2 of the current research captured absorption, peak levels, and elimination following an ethanol gavage. In birds, ethanol is typically consumed orally and similar to humans, there may be a first-pass metabolism of ethanol following an oral administration. Thus, developing a BEC profile of ethanol using a gavage route of administration may serve to take into account the possibility of the first-pass metabolism. In the current study, quail reached a peak BEC level at 1or 2 h after ethanol administration depending on the dose. The current research also revealed a sex difference in peak BECs reached following an ethanol gavage. Male quail reached a higher peak BEC compared to females. Contrary to the current experiment, female rodents often have greater peak BECs, however, this may be dependent on the route of administration and a sex difference in ADH activity (Frezza et al., 1990; Desroches et al., 1995). Similar to previous rodent research we reported BECs over time to create a BEC profile (Livy et al., 2003). BEC profiles can be used by researchers to identify the ascending and descending limbs of the BEC curve and can be used to determine whether there is evidence of acute tolerance (LeBlanc et al., 1975; Martin and Moss, 1993) and/or a biphasic effect of ethanol (Holdstock and de Wit, 1998).

Similar to previous research with rodents (Crippens et al., 1999), female quail had a smaller AUC compared to male quail, and thus female quail had less overall exposure to ethanol compared to males. Previous rodent research similarly observed a smaller AUC for female rats compared to male rats (Crippens et al., 1999). In rats, a larger AUC observed in males may depend on the tissue measured. For example, the AUC of BECs was 14% smaller in females but ethanol content was 16% smaller in brain tissue (Robinson et al., 2002). In contrast, most of the research in people has found that women have greater AUCs than men (Frezza et al., 1990; Lucey et al., 1999). This effect in women may be driven by the larger peak BECs they reach compared to males (Frezza et al., 1990), as they tend to have faster ethanol disappearance perhaps due to higher hepatic ADH activity (Vidal et al., 1990).

Contrary to our hypothesis, we did not observe a sex difference in ethanol elimination (i.e., the slope from peak to the first following time point). Both human and rat studies have shown a higher rate of ethanol elimination in females compared to males. In humans, it has been typically observed that women have a faster elimination (Cole-Harding and Wilson, 1987; Mumenthaler et al., 1999). Specifically, women had a faster rate of disappearance per volume of blood per hour (Mumenthaler et al., 1999). Similarly, female rats had a faster elimination rate from tail blood (Crippens et al., 1999).

Taken together the current studies add to the literature by examining the underlying pharmacokinetics of ethanol in an animal model previously used to study drugs of abuse. The current findings provide support for the use of quail in future pharmacological studies. The findings also provide insight into some of the pharmacokinetics of ethanol in quail that will contribute to on-going motivational and behavioral ethanol studies.
